# Factors Predicting Therapeutic Efficacy of Combination Treatment With Sitagliptin and Insulin in Type 2 Diabetic Patients: The ASSIST-K Study

**DOI:** 10.14740/jocmr2149w

**Published:** 2015-06-09

**Authors:** Masashi Ishikawa, Masahiko Takai, Hajime Maeda, Akira Kanamori, Akira Kubota, Hikaru Amemiya, Takashi Iizuka, Kotaro Iemitsu, Tomoyuki Iwasaki, Goro Uehara, Shinichi Umezawa, Mitsuo Obana, Hideaki Kaneshige, Mizuki Kaneshiro, Takehiro Kawata, Nobuo Sasai, Tatsuya Saito, Tetsuo Takuma, Hiroshi Takeda, Keiji Tanaka, Nobuaki Tsurui, Shigeru Nakajima, Kazuhiko Hoshino, Shin Honda, Hideo Machimura, Kiyokazu Matoba, Fuyuki Minagawa, Nobuaki Minami, Yukiko Miyairi, Atsuko Mokubo, Tetsuya Motomiya, Manabu Waseda, Masaaki Miyakawa, Yoshikazu Naka, Yasuo Terauchi, Yasushi Tanaka, Ikuro Matsuba

**Affiliations:** a The Study Group of the Diabetes Committee, Kanagawa Physicians Association, 3-1 Fujimi-cho, Naka-ku, Yokoyama City, Kanagawa 231-0037, Japan; b Department of Endocrinology and Metabolism, Yokohama City University, 3-9 Fukuura, Kanazawa-ku, Yokohama 236-0004, Japan; c Division of Metabolism and Endocrinology, Department of Internal Medicine, St. Marianna University School of Medicine, 2-16-1 Sugao, Miyamae-ku, Kawasaki City, Kanagawa 216-8511, Japan

**Keywords:** Type 2 diabetes, Sitagliptin, Insulin, Combination therapy, HbA_1c_, Multiple regression analysis, Body weight

## Abstract

**Background:**

It is unclear whether dipeptidyl peptidase-4 inhibitors decrease hemoglobin A_1c_ (HbA_1c_) in a glucose-dependent manner in patients on insulin therapy who have impaired insulin secretion. This study investigated factors influencing the efficacy of sitagliptin when used concomitantly with insulin to treat type 2 diabetes mellitus (T2DM) in the real-world setting.

**Methods:**

A retrospective study was conducted of 1,004 T2DM patients at 36 Japanese clinics associated with the Diabetes Task Force of the Kanagawa Physicians Association. Eligible patients had been on insulin for at least 6 months, with a baseline HbA_1c_ of 7.0% (53 mmol/mol) or higher. Baseline characteristics and laboratory data from 495 patients were subjected to multiple regression analysis to identify factors influencing the change of HbA_1c_.

**Results:**

Most patients (n = 809) received sitagliptin at a dose of 50 mg. In the 1,004 patients, HbA_1c_ decreased by 0.74% (6 mmol/mol) and body weight increased by 0.1 kg after 6 months of combination therapy. Multiple regression analysis showed that a higher baseline HbA_1c_, older age, and lower body mass index influenced the change of HbA_1c_ after 6 months. Hypoglycemic symptoms occurred in 7.4%, but none were severe.

**Conclusions:**

These results emphasize the importance of a higher HbA_1c_ at the commencement of sitagliptin therapy in patients on insulin. Glucose-dependent suppression of glucagon secretion by sitagliptin may be useful in patients with impaired insulin secretion. Sitagliptin can be used concomitantly with insulin irrespective of the insulin regimen, duration of insulin treatment, and concomitant medications.

## Introduction

Among the dipeptidyl peptidase-4 (DPP-4) inhibitors that are widely used, sitagliptin is the first to be approved for patients with type 2 diabetes mellitus (T2DM) on insulin therapy. Despite its established position, relatively few clinical studies of sitagliptin have been performed. These include a US study of patients who were predominantly on once-daily long-acting, insulin therapy [1], a Japanese study of patients on mixed insulin therapy [2], and observational studies with small populations [3, 4].

T2DM patients receiving insulin often have a longer disease duration and a reduced insulin secretion capacity. Because their baseline characteristics and insulin doses vary, clinical trials alone do not provide enough information about these patients [5]. Moreover, the effectiveness of DPP-4 inhibitors at stimulating an insulin response in patients with compromised insulin secretion on insulin therapy has not been examined. A small-scale randomized controlled trial (RCT) has shown that sitagliptin improves hemoglobin A_1c_ (HbA_1c_) and postprandial glucose levels in patients with type 1 diabetes [6]. The effect of sitagliptin in increasing glucagon-like peptide 1 (GLP-1), which results in inhibition of glucagon secretion rather than stimulation of insulin secretion, has attracted attention [7, 8].

RCTs play a significant role in evidence-based medicine. However, such trials exclude patients who do not meet the specified inclusion criteria, even though these patients are often important in the real-world clinical setting. This is particularly relevant for T2DM, since pathological conditions vary widely in patients with this disease, making it difficult to investigate all patient types in a single RCT.

In the present study, we investigated the efficacy and safety of insulin-sitagliptin combination therapy in a multicenter study to verify the usefulness of this regimen for T2DM patients on various types of insulin therapy. Our findings add some useful information to the evidence already established by RCTs.

## Methods

This retrospective study included patients receiving sitagliptin in addition to insulin from November 2011 to March 2013 at 36 diabetes clinics in Kanagawa Prefecture, Japan. Sitagliptin was started if the attending physician considered that insulin was not achieving adequate glycemic control. Glycemic control, the insulin dose, concomitant drugs, blood pressure (BP), body weight, and laboratory data were analyzed, as well as the occurrence of adverse events. Eligible patients had been on insulin for 6 months or longer, with an HbA_1c_ of 7.0% (53 mmol/mol) or higher, and had undergone follow-up for at least 6 months.

The primary endpoint was the change of HbA_1c_. To explore factors contributing to the improvement of HbA_1c_ by insulin-sitagliptin combination therapy, multiple regression analysis was performed with the following variables: age, sex, baseline body mass index (BMI), duration of insulin therapy, baseline HbA_1c_, daily insulin dose, presence/absence of diabetic neuropathy, smoking, alcohol consumption, and concomitant use of sulfonylureas (SUs), biguanides (BGs), alpha-glucosidase inhibitors (α-GIs), and thiazolidinediones (TZDs). Patients who had previously been treated with a DPP-4 inhibitor were excluded.

Results are reported as mean ± standard deviation. All analyses were carried out using SPSS version 19 software (SPSS Inc., Chicago, IL, USA). The effect of sitagliptin treatment was assessed by one-way analysis of variance and P < 0.05 was accepted as indicating statistical significance.

This study was registered with the Clinical Trials Registry (http://clinicaltrials.gov; NCT01855087) and was undertaken in accordance with the Ethical Guidelines for Clinical Studies of the Japanese Ministry of Health, Labor, and Welfare. Informed consent was not required because this was a retrospective analysis.

## Results

A total of 1,169 case record forms were collected. Then patients with sitagliptin treatment for < 6 months (n = 54), baseline HbA_1c_ < 7.0% (53 mmol/mol) (n = 81), and incomplete data (n = 30) were excluded. The remaining 1,004 patients were available for analysis and their characteristics are shown in Table 1. Based on the results of Japanese clinical studies employing sitagliptin, the drug was up-titrated in some patients from a starting dose of 50 mg to 100 mg if the former dose was not effective, while a low dose of 25 mg was selected for 65 patients with impaired renal function [9]. Among the 1,004 patients, medications other than insulin and sitagliptin were altered in 185 patients, including 144 who discontinued α-GIs or TZDs and 41 who received add-on biguanides.

**Table 1:** Characteristics of the Subjects

Age, years	63.9 ± 12.1
Sex, male/female	521/483
Body mass index	25.5 ± 4.6
Hemoglobin A_1c_, % (mmol/mol)	8.69 ± 1.31 (72 ± 12)
Fasting blood glucose, mg/dL (mmol/L)	184.3 ± 70.5 (10.23 ± 3.91)
Serum C-peptide, ng/mL (nmol/L)	1.47 ± 1.03 (0.49 ± 0.34)
Duration of diabetes, years	17.1 ± 9.0
Duration of insulin treatment, years	6.3 ± 5.6
Smoking, %	23.1
Alcohol consumption, %	22.7
Complications, %	
Diabetic neuropathy	33.6
Diabetic retinopathy	33.4
Diabetic nephropathy	38.5
Cerebrovascular disease	7.6
Myocardial infarction/angina pectoris	17.3
Arteriosclerosis obliterans	8.8
Hypertension	57.7
Dyslipidemia	61.4
Hepatic steatosis	30.0
Insulin regimen, n	
Long-acting insulin, once daily	215
Mixed insulin, twice daily	162
Three times daily, no long-acting insulin	209
Basal-bolus insulin	334
Concomitant oral medications, n	
Sulfonylureas	222
Biguanides	397
Thiazolidinediones	64
Alpha-glucosidase inhibitors	184
None	418
Dose of sitagliptin, n	
25 mg throughout	65
Up-titration from 25 mg to 50 mg	83
50 mg throughout	809
Up-titration from 50 mg to 100 mg	47

Data are mean ± standard deviation unless otherwise indicated.

**Figure 1:**
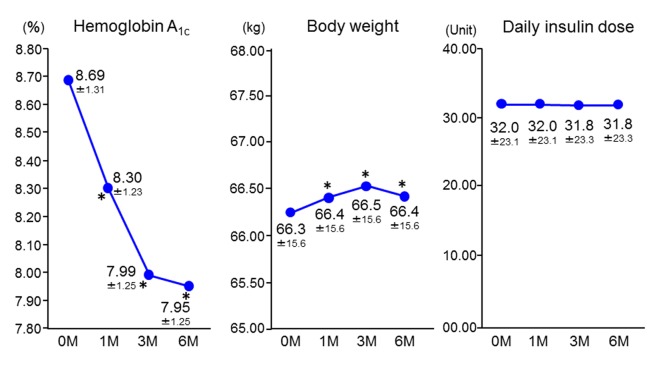
Changes in hemoglobin A_1c_, body weight, and insulin dose (n = 1,004). Data are the mean ± standard deviation. Analysis of variance vs. baseline. *P < 0.05. M: months.

After starting insulin-sitagliptin combination therapy, HbA_1c_ decreased significantly from the baseline value of 8.69±1.31% (72 ± 12 mmol/mol) to 8.30±1.23% (67 ± 11 mmol/mol), 7.99±1.25% (64 ± 11 mmol/mol), and 7.95±1.25% (64 ± 11 mmol/mol) at 1 month, 3 months, and 6 months, respectively. Body weight showed a slight, but significant, increase of 0.1 kg, while there were no changes in the doses of insulin or the other oral antidiabetic drugs (Fig. 1).

Hypoglycemic symptoms were noted in 7.4% of the subjects, but were not severe. Other adverse events included abdominal distension (0.9%) and constipation (0.4%). With regard to changes in laboratory values, there was a significant decrease of systolic BP, fasting blood glucose, glycated albumin, alkaline phosphatase, total cholesterol, high-density lipoprotein (HDL) cholesterol, and triglycerides, while there was a significant increase of uric acid and serum creatinine (Table 2).

**Table 2:** Changes in Laboratory Values

Clinical parameter	n	Baseline	1 month	3 months	6 months	Difference between baseline and 6 months
Systolic BP, mm Hg	923	130.82	130.85	130.38	130.28	
Diastolic BP, mm Hg	923	74.97	74.66^a^	74.03^a^	74.22^a^	-0.75
Fasting blood glucose, mg/dL (mmol/L)	199	181.35 (10.06)	160.62 (8.91)^a^	164.81 (9.15)^a^	160.9 (8.93)^a^	-20.45 (-1.13)
Serum C-peptide, ng/dL (nmol/L)	53	0.91 (0.30)	0.98 (0.32)	1 (0.33)	0.96 (0.32)	
Glycated albumin, %	53	23.2	21.61^a^	21.05^a^	21.03^a^	-2.17
Aspartate aminotransferase, IU/L (µkat/L)	773	27.62 (0.46)	26.41 (0.44)	27.02 (0.45)	27.56 (0.46)	
Alanine aminotransferase, IU/L (µkat/L)	770	24.66 (0.41)	24.11 (0.40)	24.79 (0.41)	24.22 (0.40)	
Gamma-glutamyl transpeptidase, IU/L	703	44.12	42.22	42.12	43.47	
Alkaline phosphatase, mg/dL	175	260.05	254.16^a^	241.82^a^	241.83^a^	-18.22
Total cholesterol, mg/dL (mmol/L)	453	194.72 (5.04)	191.56 (4.96)^a^	190.26 (4.93)^a^	191.39 (4.96)^a^	-3.33 (-0.08)
LDL cholesterol, mg/dL (mmol/L)	733	109.66 (2.84)	109.02 (2.82)	108.67 (2.81)	109.08 (2.83)	
HDL cholesterol, mg/dL (mmol/L)	794	55.62 (1.44)	55.05 (1.43)^a^	54.8 (1.42)^a^	54.72 (1.42)^a^	-0.90 (-0.02)
Triglycerides, mg/dL (mmol/L)	805	164.12 (1.85)	153.61 (1.74)^a^	153.3 (1.73)^a^	155.33 (1.76)^a^	-8.79 (-0.09)
Uric acid, mg/dL (µmol/L)	331	5.05 (300.40)	5.18 (308.13)^a^	5.32 (316.46)^a^	5.27 (313.49)^a^	+0.25 (+13.09)
Creatinine, mg/dL (µmol/L)	674	0.76 (67.18)	0.77 (68.07)^a^	0.78 (68.95)^a^	0.79 (69.84)^a^	+0.03 (+2.66)

^a^P < 0.05 (analysis of variance vs. baseline). BP: blood pressure; HDL: high-density lipoprotein; LDL: low-density lipoprotein.

Multiple regression analysis was performed to examine factors related to the change of HbA_1c_ within 6 months of starting sitagliptin. Complete baseline data on the age, sex, BMI, duration of insulin treatment, HbA_1c_, daily insulin dose, presence/absence of diabetic neuropathy, smoking, alcohol consumption, and concomitant use of SUs, BGs, α-GIs and TZDs were available for 495 patients. These characteristics of the 495 patients were similar to those of the main study population (data not shown).

A high HbA_1c_ at baseline was the strongest contributor to reduction of HbA_1c_ at 6 months after starting treatment with insulin-sitagliptin combination therapy (Table 3). In addition, BMI and age made slight, but significant, contributions to the reduction of HbA_1c_ (Table 3).

**Table 3:** Multiple Regression Analysis of Delta Hemoglobin A_1c_ After 6 Months of Insulin-Sitagliptin Combination Therapy in 495 Patients With Complete Data

Factor	Beta value	P value	Standardized beta value
Age	-0.010	0.0088	-0.124
Female sex	0.164	0.0802	0.080
Baseline body mass index	0.030	0.0048	0.136
Duration of insulin treatment, years	0.013	0.1383	0.065
Baseline hemoglobin A_1c_	-0.400	< 0.0001	-0.502
Baseline daily insulin dose	0.002	0.2401	0.057
Presence of diabetic neuropathy	0.051	0.5512	0.025
Smoking, yes	0.004	0.9694	0.002
Alcohol consumption, yes	-0.004	0.9683	-0.002
Sulfonylureas	0.191	0.0939	0.074
Biguanides	-0.162	0.0720	-0.078
Thiazolidinediones	-0.300	0.0800	-0.072
Alpha-glucosidase inhibitors	-0.096	0.3606	-0.037

We also performed multiple regression analysis on a cohort of 114 patients who had C-peptide data before commencement of sitagliptin therapy. This showed that C-peptide had no influence on the effectiveness of sitagliptin (data not shown).

## Discussion

The Diabetes Task Force of the Kanagawa Physicians Association previously conducted a large-scale trial involving more than 1,000 patients (the ASSET-K study), which established that a higher baseline HbA_1c_, shorter disease duration, and lower baseline BMI were factors contributing to reduction of HbA_1c_ in patients receiving sitagliptin combined with another oral antidiabetic drug [10-14]. However, the factors influencing the HbA_1c_-lowering effect of sitagliptin when it is used concomitantly with insulin were not fully elucidated. Therefore, we conducted the present study to investigate this issue.

We confirmed the effectiveness and safety of combining sitagliptin with insulin in a large patient population (n = 1,004) treated in the real-world setting. HbA_1c_ decreased by 0.74% (6 mmol/mol) and body weight increased by 0.1 kg, while there were no appreciable changes in the doses of insulin or other oral antidiabetic agents. In addition, there were no episodes of severe hypoglycemia, although hypoglycemic events were reported in 7.4% of the patients. In general, the formulation, dose, and frequency of insulin administration are altered if an HbA_1c_ < 7.0% (53 mmol/mol) is not achieved after a certain period. However, increasing the insulin dose may lead to adverse events such as weight gain and hypoglycemia. The present study demonstrated the effectiveness of insulin-sitagliptin combination therapy in the real-world setting. As well as improving HbA_1c_, there were significant reductions of systolic BP, fasting blood glucose, glycated albumin, alkaline phosphatase, total cholesterol, HDL cholesterol, and triglycerides, which suggest that sitagliptin may also exert a favorable effect on BP and the lipid profile, as reported in the ASSET-K study [14]. The significant increase of uric acid and serum creatinine were also in agreement with the results of the ASSET-K study, and can be explained by the fact that sitagliptin increases GLP-1, an incretin hormone with diuretic properties. In fact, a correlation between the reduction of BP and an increase of creatinine and uric acid levels has been reported [14].

We performed multiple regression analysis to identify patients who were likely to respond to addition of sitagliptin to insulin therapy. We analyzed various factors that had a potential influence on HbA_1c_ including diabetic neuropathy, smoking, and alcohol, because diabetic neuropathy may inhibit the effect of GLP-1 and patients who smoke or drink regularly are more likely to lead an unhealthy lifestyle with suboptimal diet and exercise. We identified a higher baseline HbA_1c_ as the strongest contributing factor, while a lower baseline BMI and older age were also significant. This is in agreement with the fact that older patients tended to have a lower BMI in our study. There was a positive correlation between the duration of diabetes and the duration of insulin therapy (Pearson’s correlation coefficient = 0.40), as well as between the frequency of insulin administration and the daily insulin dose (Pearson’s correlation coefficient = 0.51). Therefore, the duration of insulin therapy and the daily insulin dose were included in the multiple regression analysis as background factors, revealing that the duration of insulin therapy was not associated with the effectiveness of sitagliptin. These results support the addition of sitagliptin to insulin therapy, irrespective of the duration of insulin treatment, the daily insulin dose, concomitant medications, and the C-peptide profile. Even in patients with compromised insulin secretion, sitagliptin can still effectively stimulate insulin secretion and it also has other potential benefits. Inhibition of glucagon secretion may be involved in the efficacy of DPP-4 inhibitors for insulin-treated patients [6] and inhibition of glucagon secretion as a GLP-1 receptor agonist may be important in type 1 diabetes [15].

It has been reported that T2DM patients in East Asian countries (including Japan) have lower endogenous insulin secretion and that their BMI tends to be lower [16]. Kim et al reported that sitagliptin was particularly effective in the Japanese population [17]. In addition to the ASSET-K study, several observational studies have demonstrated the effectiveness of sitagliptin in patients with a lower BMI [11, 13, 18]. However, more research is needed to determine whether our results are related to ethnic factors, especially studies in non-Japanese populations.

There were some limitations of this study. First, it was retrospective. Second, all of the patients received sitagliptin and there was no control group without sitagliptin treatment. Third, markers for the assessment of beta-cell function (HOMA-beta) and the basal level of active GLP-1, which are factors that could influence the reduction of HbA_1c_ by sitagliptin therapy, were not measured in all patients. In fact, only 495 out of 1,004 patients were included in the detailed analysis, but these patients were considered to be reasonably representative of the entire population since their characteristics did not differ from those of the whole population (data not shown).

In conclusion, this study provided useful new information about the efficacy and safety of sitagliptin that can be added to the findings of relevant RCTs. We demonstrated that a higher baseline HbA_1c_ was associated with greater improvement of HbA_1c_ in patients given sitagliptin as add-on therapy to insulin, while other factors were not clinically relevant. This suggests that patients might be candidates for add-on sitagliptin therapy irrespective of their characteristics, insulin dose, and concomitant medications. Because a significant increase of body weight was not observed, sitagliptin may be added whenever the HbA_1c_ is not well controlled by insulin therapy.
